# Performance of Low-Dose Chest CT as a Triage Tool for Suspected COVID-19 Patients

**DOI:** 10.5334/jbsr.2319

**Published:** 2021-02-16

**Authors:** Jeroen Desmet, Charlotte Biebaû, Walter De Wever, Lesley Cockmartin, Verbraeken Viktor, Johan Coolen, Johny Verschakelen, Adriana Dubbeldam

**Affiliations:** 1 UZ Leuven, BE

**Keywords:** low-dose chest CT, COVID-19, triage, performance, pandemic

## Abstract

**Purpose:**

To investigate the role of low-dose chest computed tomography (CT) imaging in the triage of patients suspected of coronavirus disease 2019 (COVID-19) in an emergency setting.

**Materials and Methods:**

Data from 610 patients admitted to our emergency unit from March 20, 2020, until April 11, 2020, with suspicion of COVID-19 were collected. Diagnostic values of low-dose chest CT for COVID-19 were calculated using consecutive reverse-transcription polymerase chain reaction (RT-PCR) tests and bronchoalveolar lavage (BAL) as reference. Comparative analysis of the 199 COVID-19 positive versus 411 COVID-19 negative patients was done with identification of risk factors and predictors of worse outcome.

**Results:**

Sensitivity and specificity of low-dose CT for the diagnosis of COVID-19 respectively ranged from 75% (150/199) to 88% (175/199) and 94% (386/411) to 99% (386/389), depending on the inclusion of inconclusive results. On multivariate analysis, a higher body mass index (BMI), fever, and dyspnea on admission were risk factors for COVID-19 (all p-values < 0.05). The mortality rate was 12.6% (25/199). Higher age and high levels of C-reactive protein (CRP) and D-dimers were predictors of worse outcome (all p-values < 0.05).

**Conclusion:**

Low-dose chest CT has a high specificity and a moderate to high sensitivity in symptomatic patients with suspicion of COVID-19 and could be used as an effective tool in setting of triage in high-prevalence areas.

## Introduction

The diagnosis of coronavirus disease 2019 (COVID-19) pneumonia can be very challenging based on symptoms alone, because there is a great overlap with other conditions. Therefore, the diagnosis highly depends on nasopharyngeal swab reverse– transcription polymerase chain reaction (RT-PCR) testing. These tests have high specificity but rather low sensitivity, with reported false-negative rates ranging from 5–40%, depending on the assay, time of testing, and quality of the specimen [1]. In addition, it can take hours before the results are present, making it challenging as a screening tool in an emergency setting. Chest computed tomography (CT), on the other hand is readily available, and the results can be given within minutes. Typical chest CT patterns of COVID-19 pneumonia include multifocal bilateral peripheral ground-glass areas, with or without consolidation, mostly peripheral and predominantly involving lower lung lobes and posterior segments [2]. The reported sensitivity and specificity of chest CT for the diagnosis of COVID-19 vary widely (60–98% and 25–72%, respectively) [3,4,5,6].

In our hospital, we applied a low-dose (i.e., reducing the radiation burden by more than a factor of 6 as compared to standard-dose) chest CT protocol to all patients suspected of COVID-19. Low-dose chest CT was used as a complementing triage tool for patients suspected of COVID-19 with symptoms worse enough for admission to the hospital. When clinical and CT findings were suggestive for COVID-19 pneumonia, the results of the RT-PCR test were not awaited. These patients were admitted to dedicated COVID-19 wards. As such, the capacity in our emergency room wasn’t overwhelmed during the peak of the first outbreak wave. For inconclusive chest CTs, the RT-PCR test was awaited, and alternative diagnoses were excluded.

This single-institution study aimed at evaluating retrospectively the diagnostic values of low-dose chest CT for the diagnosis of COVID-19 pneumonia as compared to consecutive nasopharyngeal swab and bronchoalveolar lavage (BAL) RT-PCR tests, if available.

## Materials and Methods

### Patient Population and Study Design

**Figure 1:**
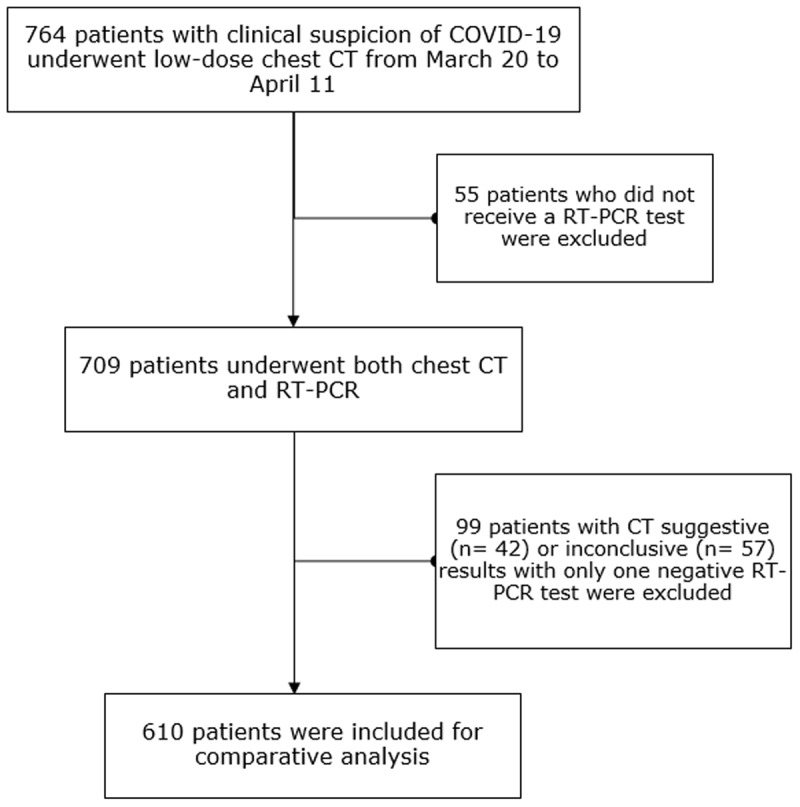
Flowchart of patient inclusion. **Abbreviations:** n = number of patients, RT-PCR = reverse transcription polymerase chain reaction.

**Figure 2:**
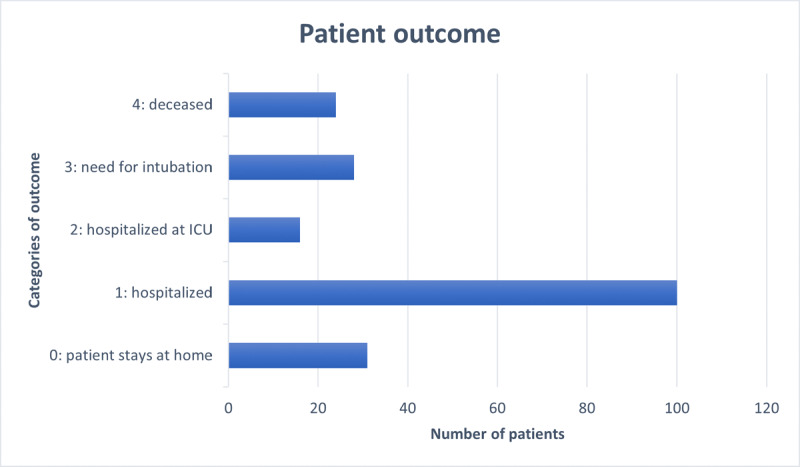
Patient outcome of the 199 COVID-19 positive cases.

From March 20 to April 11, 2020, all patients suspected of COVID-19 infection underwent a low-dose chest CT in our institution. Of the 764 consecutive symptomatic patients, 709 had a nasopharyngeal swab test. Patients with a single negative RT-PCR, but a chest CT suggestive or inconclusive, were not included to avoid bias due to false-negative RT-PCR [1]. In patients with multiple RT-PCR or bronchoalveolar lavage (BAL) during hospitalization, the presence of one positive test result overruled previous negative RT-PCR (see ***Figure 1***). Patient characteristics, imaging, and lab results were extracted from the medical records. Patient outcomes were divided into five categories based on the need for hospitalization, intensive care, and intubation (***Figure 2***). The ethical committee of our hospital approved this retrospective study.

### Imaging Technique and Interpretation

All patients underwent scanning with SOMATON definition flash (Siemens Healthcare, Erlangen, Germany). A low-dose chest CT protocol, without intravenous contrast agent injection, was used. The tube voltage and tube current were set depending on the weight of the patients, that is, 80 kV and 30 mAs for patients < 50 kg, 120 kV, and 20 mAs for 50–80 kg and 140 kV and 28 mAs for > 80 kg. Tube current modulation was turned off. All other exposure parameters, such as pitch factor 1.2, rotation time 0.5 s, and collimation 128 × 0.6 mm, were set constant for all patients. The median dose-length product for our study dataset was 53mGy.cm, that is, approximately six times less than for a standard-dose chest CT.

**Figure 3:**
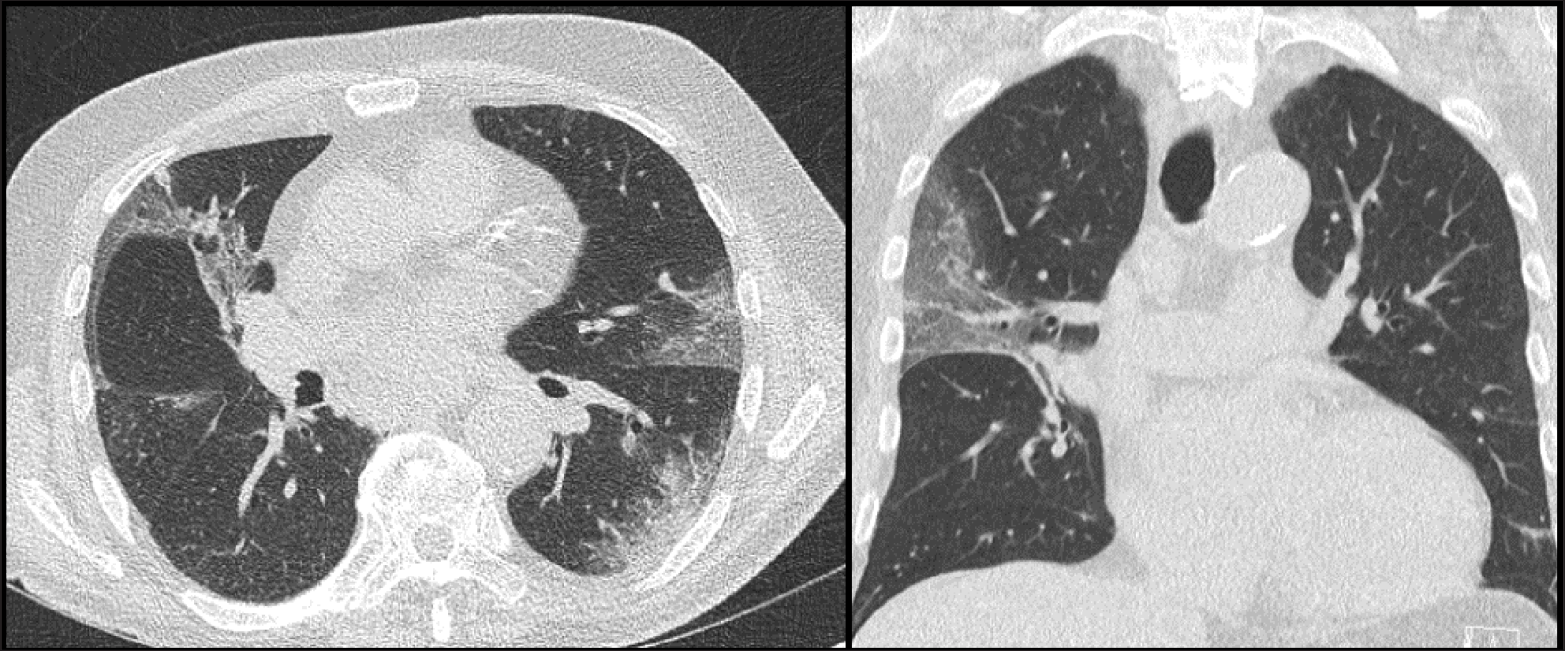
Low-dose chest CT with findings suggestive for COVID-19 pneumonia, showing multifocal, bilateral peripheral ground-glass opacities and consolidations.

**Figure 4:**
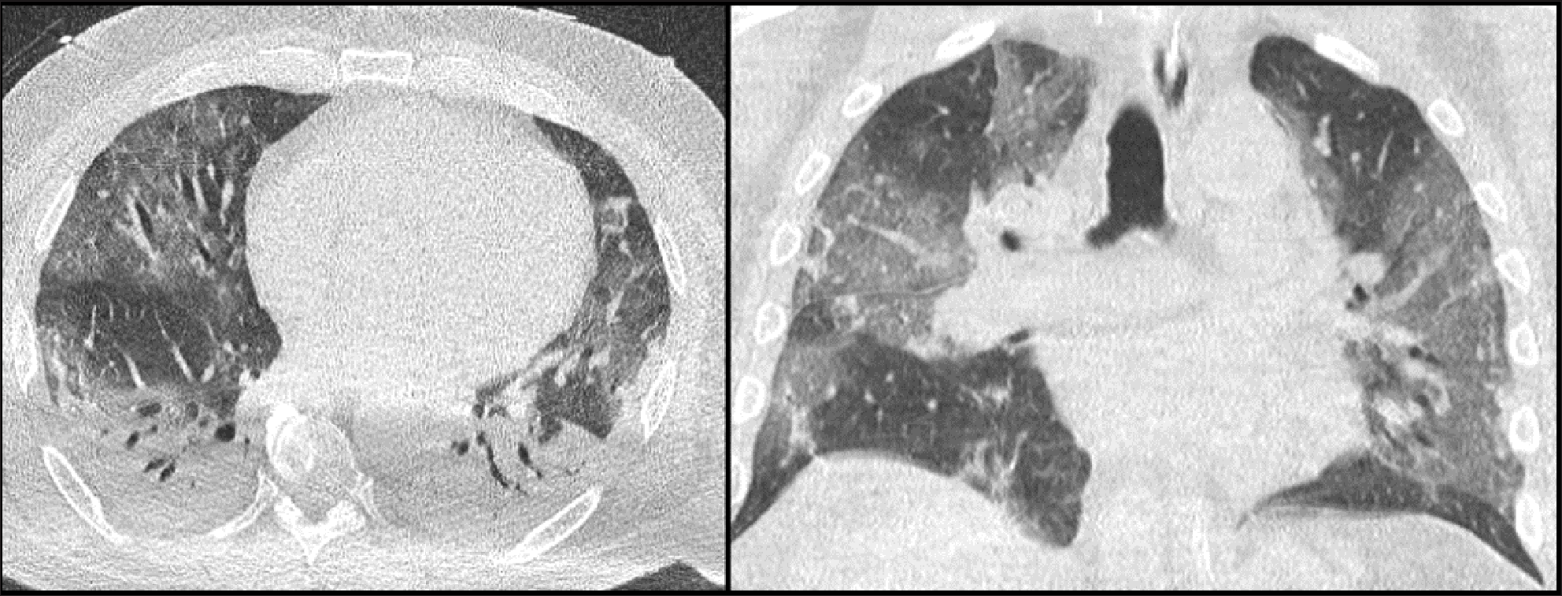
Low-dose chest CT with findings inconclusive for COVID-19 pneumonia, showing bilateral ground-glass opacities with peripheral-central distribution as a typical finding and bilateral pleural fluid as an atypical finding. RT-PCR and BAL turned out positive for COVID-19 in this patient.

**Figure 5:**
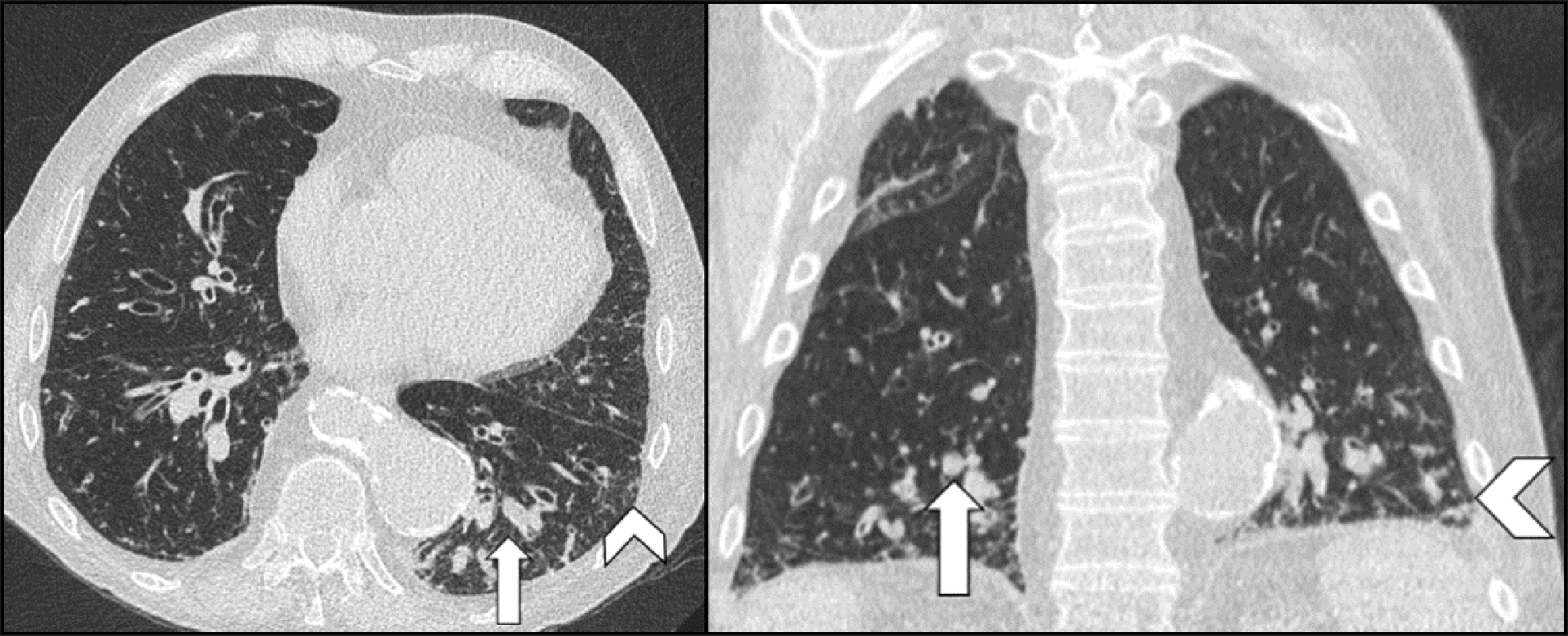
Low-dose chest CT of a patient with known chronic obstructive pulmonary disease with findings inconsistent (negative) for COVID-19 pneumonia. No typical findings for COVID-19 were seen. Bronchiectasis with mucus plugs (arrows) and discrete zones of tree-in-bud (arrowheads) are shown. RT-PCR was negative for this patient. Patient was treated for acute exacerbation.

Reports of CTs were standardized, with the description of typical and atypical findings. Consistent findings included the presence of multifocal ground-glass opacities, with peripheral or peripheral-central distribution. Inconsistent findings included the presence of tree-in-bud pattern, nodules, pleural fluid, enlarged lymph nodes, and the presence of cavitation [2,3,4,5,6,7]. Patients were divided into three categories: suggestive, inconclusive, or inconsistent for COVID-19 pneumonia. The CT was considered suggestive for COVID-19 pneumonia if typical findings were present in the absence of atypical findings (***Figure 3***). The term “inconclusive for COVID-19 pneumonia” was used for patients with findings that have been reported with COVID-19 pneumonia but not specific enough for a confident radiological diagnosis. For example, diffuse ground-glass opacities without clear distribution or with lung collapse, which makes affirmation or exclusion of COVID-19 pneumonia virtually impossible [6,7]. Additionally considered inconclusive CTs were those showing mixed typical and atypical findings (***Figure 4***). The CT was regarded as inconsistent for COVID-19 pneumonia if there were no abnormalities or if there were atypical findings, with none of the typical findings (***Figure 5***).

### Statistical Analysis

Unpaired group comparisons between COVID-19 positive and negative cases were performed for the following patient-related parameters: age, gender, smoking habits, body mass index (BMI) and clinical symptoms. Chi-square test was used for categorical variables and the Mann-Whitney U test for continuous variables. Furthermore, risk factors were identified by binary logistic regression with COVID-19 diagnostic ground truth (positive or negative) as a dependent variable and each other patient parameter, that is, age, gender, smoking habits, BMI, and clinical symptoms) as an independent variable. Predictors of patient outcome were identified by ordinal logistic regression with patient outcome (i.e., hospitalized, admitted at intensive care, intubated, or deceased) as a dependent variable and each other patient parameter as an independent variable (only for the COVID positive patients). Some of our significant continuous variables were rescaled so that the clinical meaning of the odds ratio for that predictor might become clearer. BMI was divided by 10, age was divided by 10, C-reactive protein (CRP) levels were divided by 10, and D-Dimers were divided by 1000. Using RT-PCR and BAL as a reference diagnostic technique, sensitivity, specificity, positive predictive value (PPV), negative predictive value (NPV), and accuracy of chest CT were calculated. The IBM Statistical Package for Social Sciences (SPSS version 13, IBM Corp., Armonk, NY, USA) was used; statistical significance level was 0.05.

## Results

### Patient Population

Our study population consisted of 610 patients (***Figure 1***) (352 males, 258 females; mean age 65.25 years). On imaging, the findings of 153 patients (25%) were suggestive of COVID-19 pneumonia, inconsistent in 410 (67%) and inconclusive in 47 (7.7%). Based on nasopharyngeal or BAL RT-PCR results, 199 patients (33%) were considered COVID-19 positive and 411 (67%) COVID-19 negative. Patients who tested positive for COVID-19 had a significant higher BMI (27.3 versus 25.7) and were more likely to have fever, cough, dyspnea, or myalgia at admission compared to the COVID-19 negative patients (all p-values < 0.05) (***Table 1***).

**Table 1:** Characteristics of the 610 patients included.


	COVID-19 POSITIVE	COVID-19 NEGATIVE	P-VALUE

**Number**	199	411	

**Age** (years)	65.27	65.23	0.816

**Gender**			

Male	120 (60%)	232 (56%)	

Female	79 (40%)	179 (44%)	0.366

**BMI** (kg/m^2^)			

BMI	27.25	25.73	0.004

**Smoking habit**			

Active	10 (5%)	72 (17.5%)	<0.001

Former	69 (34.8%	152 (37%)	

Never	96 (48.2%)	153 (37.2%)	

Unknown	24 (12.1%)	34 (8.3%)	

**Symptoms**			

Fever	146 (73.4%)	182 (44.3%)	<0.0001

Cough	118 (59.3%)	199 (48.4%)	0.012

Dyspnea	147 (73.9%)	239 (58.1%)	<0.0001

Myalgia	24 (12.1%)	29 (7.1%)	0.04


**Abbreviations:** N = number, χ^2^ = chi-square test.

### Patient Outcomes

Of the 199 COVID-19 positive patients, 31 (15.6%) were sent home and 168 (84.4%) were admitted to the hospital. Among this group, 48 patients needed high supportive care, of which 31 had to be intubated. Twenty-five patients out of 199 (12.6%) died.

The mean duration of hospitalization was 11.8 days (standard deviation 11.0, range 0–47). There was a moderate correlation (r_s_ = 0.62) between increasing days of hospital stay and worse patient outcome. A complete overview of the patient outcomes can be seen in ***Figure 2***.

On multivariate analysis, a higher BMI, and presentation with fever or dyspnea were significant risk factors for COVID-19 pneumonia (***Table 2***).

**Table 2:** Risk factors for COVID-19 in our study population (n = 610).


	UNIVARIATE ANALYSIS, RISK FACTOR FOR COVID-19 OR (P VALUE)	MULTIVARIATE ANALYSIS, RISK FACTOR FOR COVID-19 OR (P VALUE)

**Age**	1.000 (0.980)	NM

**Gender** (male)	1.172 (0.367)	NM

**BMI**/10 (kg/m^2^)	1.640 (0.002)	1.565 (0.01)

**Never smoked**	4.525 (<0.0001)	NM

**Symptoms**		

Fever	3.466 (<0.0001)	3.450 (<0.0001)

Cough	1.552 (0.012)	1.263 (0.237)

Dyspnea	2.034 (<0.0001)	2.122 (<0.0001)

Myalgia	1.807 (0.042)	1.217 (0.556)


**Abbreviations:** NM = not measured. OR = odds ratio. BMI = Body Mass Index.

Higher age and high levels of CRP and D-dimers were significant predictors of worse patient outcome for the 199 COVID-19 positive patients (***Table 3***).

**Table 3:** Predictors of worse outcome in COVID-19 positive cases (n = 199).


	UNIVARIATE ANALYSIS, PREDICTOR OF WORSE OUTCOME OR (P VALUE)	MULTIVARIATE ANALYSIS, PREDICTOR OF WORSE OUTCOME OR (P VALUE)

**Age**/10	1.357 (<0.0001)	1.309 (0.029)

**Gender** (male)	1.980 (0.014)	1.667 (0.165)

**Smoking**	1.489 (0.519)	NM

**BMI** (kg/m^2^)	1.040 (0.117)	NM

**Symptoms**		

Fever	1.432 (0.236)	NM

Cough	1.254 (0.403)	NM

Dyspnea	1.912 (0.036)	0.902 (0.800)

Myalgia	0.471 (0.072)	NM

**Lab results**		

Total WBC (count * 10^9/l)	1.179 (<0.0001)	1.054 (0.305)

Lymphocytes (count * 10^9/l)	1.085 (0.603)	NM

Neutrophils (count * 10^9/l)	1.254 (<0.0001)	1.034 (0.689)

CRP levels/10 (mg/dl)	1.093 (<0.0001)	1.057 (0.024)

D-dimers/1000(ng/ml)	1.255 (0.004)	1.234 (0.005)


**Abbreviations**: OR = odds ratio, BMI = body mass index, WBC = white blood cell count, NM = not measured, CRP = C-reactive protein.

### CT Diagnostic Value for COVID-19

Forty-seven (7.7%) of the 610 included patients had inconclusive chest CT. All of these patients were clinically reevaluated, combined with consecutive nasopharyngeal swab and BAL PCR tests. BAL was performed in 27 patients, positive for COVID-19 in 5 cases and negative in 22. Eventually, 25 of the initially inconclusive patients (53.2%) were considered COVID-19 positive, whereas 22 were regarded as COVID-19 negative.

Diagnostic performance of CT was first calculated by not taking into account the patients with CT inconclusive results, resulting in a total of 563 patients. The sensitivity and specificity of chest CT for COVID-19 was then 86% (150/174) and 99% (408/411) respectively.

When considering inconclusive CT as suggestive for COVID-19, we obtained a sensitivity and specificity of respectively 88% (175/199) and 94% (386/411). If we consider inconclusive CT results as negative for COVID-19, the sensitivity and specificity were respectively 75% (150/199) and 99% (408/411) (***Table 4***).

**Table 4:** Summary of diagnostic performance of chest CT for the diagnosis of COVID-19 according to the allocation of patients with inconclusive results (n = 47).


	INCONCLUSIVE NOT INCLUDED	INCONCLUSIVE COUNTED AS SUGGESTIVE FOR COVID-19	INCONCLUSIVE COUNTED AS INCONSISTENT FOR COVID-19

N	563	610	610

Sensitivity	86% (150/174)	88% (175/199)	75% (150/199)

Specificity	99% (386/389)	94% (386/411)	99% (408/411)

Accuracy	95% (536/563)	92% (561/610)	91% (558/610)

PPV	98% (150/153)	88% (175/200)	98% (150/153)

NPV	94% (386/410)	94% (386/410)	89% (408/457)


**Abbreviations:** PPV = positive predictive value, NPV = negative predictive value.

## Discussion

Our study shows a moderate to high sensitivity (75–88%) and a very high specificity (94–99%) of low-dose chest CT for the diagnosis of COVID-19. In comparison, in a report by Ai et al. of 1014 cases, the sensitivity and specificity of chest CT was respectively 97% (580/601) and 25% (105/413) [3]. In another series of 460 patients, sensitivity and specificity of respectively 77.6% (104/134) and 69% (225/326) were reported [5]. The higher specificity in our study can be partly due to our different approach in defining a COVID-19 positive case. In previous studies, the RT-PCR test was used as the only reference in comparative analysis, ignoring the high rate of false negatives [1,7]. We decided to use a definition of COVID-19 based on consecutive PCR testing and BAL, if available. Therefore, false positive rate of imaging is expected to be lower. Another reason for the high specificity in our results is the period of acquisition of our data. In March and April of 2020, a large number of patients were infected by COVID-19 in our country. An alternative diagnosis (e.g., organizing pneumonia or another virus causing pneumonia) in a patient presenting with suspicious clinic and radiologic findings for COVID-19 pneumonia will be low.

The moderate sensitivity in our study population can be explained by the retrospective study design. Not all of our patients needed a low-dose COVID-chest CT. In some patients who had only mild symptoms and consequently did not require hospital admission, CT imaging was still ordered. Furthermore, after the initial peak, and with increasing non-COVID activity, more and more patients were sent for chest CT as a screening tool (e.g., before major surgery).

When reporting, we used CT inconclusive results to deal with the uncertainty in imaging of COVID-19 pneumonia. This is in line with current recommendations for reporting, which either advise using a scoring system (CO-RADS) or a category of “indeterminate appearance” [8]. The use of these categories resulted in more true positives and true negatives and was found to be very practical in our daily practice. A suggestive CT can confidently result in action of the clinician, without the need to wait for the result of the RT-PCR test. In an inconclusive report, the results of RT-PCR test must be awaited and in selective cases, a BAL could be performed. Also, in our institution, a “COVID-maybe” unit was organized for these patients with inconclusive or conflicting results.

Severely symptomatic COVID-19 patients will often undergo multiple imaging studies during the course and recovery of their illness, reinforcing the need for a low as reasonably achievable radiation dose protocol [9]. Replacing conventional CT with low-dose CT results in reliable detection of COVID-19 pneumonia, as stated in previous studies [10]. Therefore, a low-dose chest CT for all the patients was used, with the same CT parameters as in previous large studies in the setting of lung cancer screening [11]. It is currently unknown whether further dose reduction will affect diagnostic performance.

### Limitations

To partly solve the problem with the high rate of false negative RT-PCR tests in diagnosing COVID-19 pneumonia, we excluded 99 patients with a single negative RT-PCR but a chest CT that was suggestive or inconclusive. Forty-two of these patients had a CT that was suggestive for COVID-19 pneumonia, but an RT-PCR negative result. This could have affected our rate of false positives. However, all these patients were treated as being COVID-19 positive in our hospital without the need for further confirmation with consecutive RT-PCR testing or BAL. In our opinion, these “false-positive” CTs are due to the low sensitivity of RT-PCR, rather than the underperformance of chest CT [1]. Further prospective studies with a more performant reference standard are necessary to confirm our results [1].

## Conclusion

Low-dose chest CT has a moderate to high sensitivity and a high specificity for the diagnosis of COVID-19 pneumonia and could be used as a useful complementary tool in the triage of patients with symptoms suggestive of COVID-19.
